# Time-course analysis of genome-wide gene expression data from hormone-responsive human breast cancer cells

**DOI:** 10.1186/1471-2105-9-S2-S12

**Published:** 2008-03-26

**Authors:** Margherita Mutarelli, Luigi Cicatiello, Lorenzo Ferraro, Olì MV Grober, Maria Ravo, Angelo M Facchiano, Claudia Angelini, Alessandro Weisz

**Affiliations:** 1 Department of General Pathology - Second University of Napoli, Napoli, Italy; 2 Institute of Food Sciences, National Research Council (ISA-CNR), Avellino, Italy; 3 AIRC Naples Oncogenomics Center, Napoli, Italy; 4 Institute of Applied Calculus, National Research Council (IAC-CNR) Napoli, Italy

## Abstract

**Background:**

Microarray experiments enable simultaneous measurement of the expression levels of virtually all transcripts present in cells, thereby providing a ‘molecular picture’ of the cell state. On the other hand, the genomic responses to a pharmacological or hormonal stimulus are dynamic molecular processes, where time influences gene activity and expression. The potential use of the statistical analysis of microarray data in time series has not been fully exploited so far, due to the fact that only few methods are available which take into proper account temporal relationships between samples.

**Results:**

We compared here four different methods to analyze data derived from a time course mRNA expression profiling experiment which consisted in the study of the effects of estrogen on hormone-responsive human breast cancer cells. Gene expression was monitored with the innovative Illumina BeadArray platform, which includes an average of 30-40 replicates for each probe sequence randomly distributed on the chip surface. We present and discuss the results obtained by applying to these datasets different statistical methods for serial gene expression analysis. The influence of the normalization algorithm applied on data and of different parameter or threshold choices for the selection of differentially expressed transcripts has also been evaluated. In most cases, the selection was found fairly robust with respect to changes in parameters and type of normalization. We then identified which genes showed an expression profile significantly affected by the hormonal treatment over time. The final list of differentially expressed genes underwent cluster analysis of functional type, to identify groups of genes with similar regulation dynamics.

**Conclusions:**

Several methods for processing time series gene expression data are presented, including evaluation of benefits and drawbacks of the different methods applied. The resulting protocol for data analysis was applied to characterization of the gene expression changes induced by estrogen in human breast cancer ZR-75.1 cells over an entire cell cycle.

## Background

Estrogens (E2) are key regulators in many biological processes, along with a highly recognized role in breast cancer where they control key cellular functions by diffusing through the cell membrane and interacting with the estrogen receptors (ERs), transcription factors which play an important role in controlling multiple cellular processes mainly via changes in the expression of selected genes [1-3]. Complexity of the cellular responses to estrogen and their receptors can ideally be investigated only with comprehensive analytical approaches, including in particular gene expression profiling with microarrays [4,5]. These technologies allow to assess at genome-wide scale changes in gene activity resulting, for example, from hormonal and pharmacological treatments or pathological and divergent physiological conditions. As changes in gene expression are driven by a dynamic process, the influence of time should not be neglected, but the use of this technique to study kinetics of gene expression changes has not been fully exploited yet. Indeed, few statistical methods are available which enable to fully evaluate time series. Most of the methods to identify differentially expressed genes adapt classical techniques originally designed for static experiments. This ‘static’ approaches have the disadvantage of not taking into account temporal relationship among samples, leading to results that are often invariant under permutation of the values representing different time points, thus ignoring the biological causality which can be inferred from the temporal response. They do not accurately consider the existing temporal structure in the data which can have as consequence a falsely calculated significance of the genes.

For example, the popular microarray analysis package SAM (Significance Analysis of Microarrays) [6] was recently adapted to handle time course data, by considering different time points as distinct groups; the ANOVA [7] approach can also be applied to time course experiments by treating the time variable as a particular experimental factor and other methods [8-10], including the *limma* package [11] which uses linear models, follow similar approaches.

On the other hand, most classical time series algorithms, mainly used for signal processing, are quite rigid, including requirement of a large number of time-points, uniform sampling intervals and absence of replicated or missing data-points, which microarray experiments rarely meet.

Recently the time variable is starting to be much more considered in the analysis of regulation of gene expression, leading to new developments in the area of analysis of time-course microarray [12,13]. Due to the constraints in microarray data structure, however, the problem of detecting and estimating gene expression profiles becomes extremely challenging and robust statistical methodologies are still missing. On the other hand, very few large scale comparisons are available in order to illustrate benefits and drawbacks of current methodologies.

With the aim of setting up a workflow adapted for time course experiments, we tested the available methods tailored for time series analysis and established an analysis protocol to be used in subsequent experiments. The first method we considered introduces the time variable through a gene expression response curve which is expanded over the polynomial or B-spline basis with the coefficients estimated by the least squares procedure [14] (implemented in the software EDGE - Extraction of Differential Gene Expression [15]). The second method uses a novel multivariate empirical Bayes approach to rank genes in the order of interest from longitudinal replicated microarray time course experiments [16] (implemented in the Bioconductor [17] package *timecourse*). However, this last method does not consider time curves from a functional point of view, neither provides any cut-off to select statistically significant genes. The third method is a functional Bayesian approach in which each gene expression temporal profile is estimated globally by expanding it over an orthogonal basis [18] (implemented in the software BATS - Bayesian Analysis of Time Series [19]).

Our aim here, rather than to propose new methodologies, is to provide a detailed comparison of different methods which can be used as suitable protocol for analysis of time course gene expression data from microarray experiments.

## Methods

### Cell-lines cultures and array hybridizations

Human estrogen-responsive breast cancer cells (ZR-75.1) cultured in steroid-free medium for 4 days were stimulated with a mitogenic dose (10nM) of 17β-estradiol and RNA was extracted before or after 1, 2, 4, 6, 8, 12, 16, 20, 24, 28 and 32 hours hormonal stimulation. Cells were collected from multiple parallel cultures and pooled before RNA extraction as described before [4]. Hybridization reactions were performed with Illumina Human WG-6 BeadChips following manufacturer's protocols, in duplicate for each sample, except the reference sample (before stimulation - 0h) which was in quadruplicate and the 4h sample in triplicate. In the Illumina arrays the oligonucleotides are attached to microbeads which are then put onto microarrays using a random self-assembly mechanism [20]. Also, due to the small dimension of the beads, each bead-type (representing one probe for a total of 46713 sequences) is present in a number of the order of ≈ 30-40 copies, thus providing an internal technical replication that other platforms usually lack. In the present paper, we use the term ‘probe’ and ‘bead’ indifferently, since in each case we use as signal the mean value of each bead population of signals present on the array.

The complete datasets will be submitted to the public repository of microarray data ArrayExpress upon publication.

### Pre-processing

Five different normalization algorithms were applied on data, three of them present in the chip manufacturer's analysis software BeadStudio and two of them performed using R/Bioconductor statistical environment [17,21]. The **average** method simply adjusts the intensities of each signal so that the average signal of each array becomes the same. The **rank invariant** is very similar, the only difference is that the scaling factor is calculated only on a subset of rank-invariant genes and not on all genes [22]. The **cubic spline** is the only non-linear method present in the BeadStudio software, similar to an existing algorithm [23] and described in the software manual [22]. The **quantile** method [24] acts to uniform the quantile distribution of each array signal population and is widely used as standard in single-channel arrays [25]; it is available through the R/Bioconductor packages *affy *[26] or *limma *[11] and many other popular analysis software. **Lumi**[27] is a new method especially designed for Illumina BeadChips, based on a modification of the variance stabilizing normalization algorithm [28] to make use of the bead standard deviation associated to each signal, only available in this microarray platform.

After normalization, probe signals were checked for detection against negative controls with a BeadStudio internal algorithm and missing values were introduced to replace signals under the detection limit. Probes in which the reference sample had less than 3 out of 4 detected signals were filtered out. Then *log*2 transformation was applied on data, except in the case of lumi which uses its own variance stabilizing transformation. Ratios of each signal against the average reference signal were calculated and probes with more than 15% missing values of the resulting time series were filtered out.

### Time series analysis

The following sections contain a brief description of the methods used in this paper to perform the statistical analysis of a microarray experiment made in the course of time. For a detailed description of each method, we refer the reader to each method's reference. Some preliminary considerations are however necessary: the number of time points *t*^(*j*)^, *j* = 1,…, *n* at which each sample is taken is relatively small (*n* ≈ 10) and the experimental design is not generally regular, with very few replicates at each time point (*k_i_*^(*j*)^ = 0,…, *K*, *K* = 1, 2 or 3); on the other hand a very large number of genes (*N* ≈ 10^4^) are simultaneously measured, some data points might be missing due to technical error and the noise is usually not gaussian.

#### Sliding window analysis

We first extracted a list of differentially expressed genes at each time-point using the internal DiffScore test of BeadStudio software [22] by using thresholds of different stringency (a DiffScore of 20 and 30, corresponding respectively to a *p*-value of 0.01 and 0.001 of the underlying statistical test). We denoted as ‘differentially expressed’ genes those which were selected at least in three consecutive time-points. The limits of this procedure are the lack of statistical formalization and the fact that the fixed window does not account for irregularly spaced grid assigning to all points the same weight.

#### EDGE

The method proposed in Storey *et al*. [14] apply both to longitudinal and independent data. For each gene the effect of the treatment is modeled as a mathematical function and expanded over the polynomial or *p*-dimensional B-spline basis [*s*_1_ (*t*),…, *s_p_*(*t*)]. In our case data are not truly longitudinal since the biological source is a cell line, cultured in parallel, under identical and controlled conditions, hence the method is applied in its simplified version.

Let $z_{i}^{j,k}$ be the relative expression level of the gene *i* in the *k^th^* replicates at the *j^th^* time point *t*^(*j*)^ where there are *i* = 1,…, *N* genes and *j* = 1,…, *n* time points, $k\;=\;1,...,\;k_{i}^{\left(j\right)}$ replicates for time point. The relative observed gene expression values are then modeled by

$$z_{i}^{j,k}\;=\;\mu_{i}\left(t^{\left(j\right)}\right)\;+\;\zeta_{i}^{j,k}$$

where µ_*i*_(*t*^(*j*)^) is the (unknown) relative expression time curve for gene *i* evaluated at time *t*^(*j*)^ and can be written in terms of a *p*-dimensional linear basis [*s*_*1*_(*t*),…, *s_p_*(*t*)]:

$$\mu_{i}\left(t\right)\;=\;\beta_{0,i}\;+\;\beta_{i,1}s_{1}\left(t\right)\;+\;\beta_{i,2}s_{2}\left(t\right)\;+\;...\;+\;\beta_{i,p}s_{p}\left(t\right)$$

where, β_0,*i*_ is the intercept term, *p* is the same for all genes (it is assumed to be known and in practice it is preliminarily estimated from the data or it can be provided by the user), and $\zeta_{i}^{j,k}$ are modeled as independent random variables with mean zero and gene dependent variance $\sigma_{i}^{2}$. Under this setup the interest is to test the null hypothesis *H*_0,*i*_ that μ*i*(*t*) = 0 against the alternative *H*_1,*i*_ formulated under the general parametrization μ*i*(*t*) = β_*i*,1*s*1_(*t*) + β*i*, 2*s*2(*t*) + … + β_*i*,*p*_*s*_*p*_(*t*) with some non zero coefficients. To assess differentially expressed genes, the goodness of model fit under the null hypothesis is compared to that under the alternative hypothesis, by calculating for gene *i* a *F* statistic similar to the one used in ANOVA:

$$F_{i}\;=\;\frac{SS_{i}^{0}\;-\;SS_{i}^{1}}{SS_{i}^{1}},$$

where $SS_{i}^{0}$ is the sum of squares of the residuals obtained from the null model, and $SS_{i}^{1}$ from the alternative model. However, Storey *et al*. [14] do not impose assumption of normality: the distribution of these statistics is treated as unknown and studied via bootstrap [29], which may require high computational cost. Finally, to account for the multiplicity of comparisons, the most significant curves are selected by controlling *q*-values using an FDR-like procedure [30].

This method is implemented in the user-friendly software EDGE [15]. We used the software with default parameter setting (increasing the number of iterations to 1000 in order to reduce the problem of the granularity of the *p*-values and to obtain more stable lists) and *q*-value thresholds of 0.01 and 0.001. The ‘*K* nearest neighbor’ (KNN) method [31] is provided to impute missing values, since the method itself does not account for missing data. In order to separate the effect of the method from the procedure to impute the missing values, we repeated the analysis both by filtering out all the genes with missing observations and by using the KNN method to impute them.

#### timecourse

This method applies the novel multivariate empirical Bayes approach described in Tai *et al*. [16] to rank genes in the order of interest from longitudinal replicated microarray time course experiments. Similarly to Storey *et al*. [14], *timecourse* can be applied both to the ‘one-sample’ and ‘two-sample’ case, however in the last case it is applicable only to data sets with identical time grids. On the other hand, differently from Storey *et al*. [14] where both longitudinal and independent sampling designs are accounted or from Angelini *et al*. [18] where only the independent sampling is considered, this method is designed for data where replicates are biologically meaningful, for example when a full series of time-points is drawn from the same individual (i.e., truly longitudinal). Indeed, biological samples are treated under the ‘fixed effects’ rather than the ‘random effects’ design model. Hence, since in this context one replicate is a full time curve (i.e, vector of size *n*), missing data are not allowed and the same number of arrays is required at any time point. On the other hand, different number of replicates are allowed between different genes.

For each gene *i* and individual *k* the *n*-dimensional vector of observations $z_{i}^{k}\;=\;{\left(z_{i,1}^{k},\;\ldots ,\;z_{i,n}^{k}\right)}^{T}$ on the grid *t*^(1)^,…,*t*^(*n*)^ is assumed to be conditionally independently drawn from a multivariate n-variate normal distribution with unknown mean μ*_i_* and covariance matrix Σ_*i*_, i.e.,

$$z_{i}^{k}|\mu_{i},\;\sum_{i}\;∼\;N_{n}\left(\mu_{i},\;\sum_{i}\right)$$

The method only seeks a statistic for ranking genes in the order of evidence against a null hypothesis and does not attempt to find a threshold to select the significant genes. The null hypothesis corresponding to a gene mean expression level being zero is defined as *H*_0,*i*_: μ_*i*_ = 0, Σ_*i*_ > 0 and the alternative as *H*_1,*i*_ : μ_*i*_ ≠ 0, Σ_*i*_ < 0. An *N*-dimensional indicator random variable *I* is defined to reflect the status of the genes:

$$I_{i}\;=\;\{\begin{array}{cc} 1, & \text{if}\;H_{1,i}\;\text{is}\;\text{true}\\ 0, & \text{if}H_{0,i}\;\text{is}\;\text{true} \end{array}$$

with a Bernoulli distribution with success probability ω, 0 < ω < 1. The multivariate hierarchical Bayesian model is built by elicitating the following priors:

$$\begin{array}{l} \mu_{i}\;|\;\sum_{i},\;I_{i}\;=\;1\;∼\;N_{n}\;\left(0,\;\eta^{-1}\;\sum_{i}\right)\;\text{and}\;\mu_{i}\;|\;\sum_{i},\;I_{i}\;=\;0\;∼\;\delta \;\left(0,...,0\right)\\ \;\;\;\;\;\;\;\;\;\;\;\;\;\;\;\;\;\;\;\;\;\;\;\sum_{i}\;∼\;{\text{Inv-Wishart}}_{\nu }\;\left({\left(\nu \Lambda \right)}^{-1}\right) \end{array}$$

where η > 0 is a scale parameter, ν > 0 and ν Λ < 0 are the degrees of freedom and scale matrix, respectively. Since conjugate priors are elicited on the unknown parameters, all computations for the posterior distributions and the form of the statistics are carried out in a analytical form. Moreover, the hyper-parameters, whose amount however increases with the number of time points, can be estimated from the data.

Finally, the Hotelling *T*^2^-statistic is calculated and used to rank genes when the same number of replicates are available for all genes, while the *M B*-statistics is used when the number of replicates is not equal for all genes [16]. For further details on the statistics and on parameters estimation, we refer the interested reader to the original reference. Here we only note that, due to the way the data model was conceived, the quantitative information about the time measurements is not explicitly used by this method. The method is implemented in the *timecourse* R/Bioconductor package [32]. We applied the method using the first two replicates per time point, since the number of replicates has to be the same along the time curve. Also since missing values are not allowed, we repeated the analysis both by filtering out all the genes with missing observations and by using a KNN algorithm implementation present in R [33].

#### BATS

BATS (Bayesian Analysis of Time Series) software [19] is a newly-developed user friendly tool which implements the functional Bayesian approach described in Angelini *et al*. [18]. Although independently developed, the method appears to be a compromise between EDGE and *timecourse*. Indeed, similarly to EDGE, the method treats records as functional data, thus preserving causality and taking into account the temporal nature of data. Similarly to *timecourse*, the Bayesian approach is applied in the method at all stages of analysis, but the priors are elicited on the space of the function coefficients, hence the time variable enters in the model in explicit form trough the design matrix.

BATS is designed for data consisting of the records on *N* genes and describing the difference in gene expression levels between treatment and control in a context of independent sampling time course experiment. A gene record is defined as a vector of size *M_i_*, containing all the measurements available for gene *i*. Each record is modeled as a noisy measurement of a function μ_*i*_(*t*) at a time point *t*^(*j*)^ ∈ [0, *T*] as in equation (1) where for each gene *i*, its expression profile μ_i_(*t*) is expanded into series over some standard orthonormal basis [φ_0_(*t*) φ_1_(*t*) ··· φ_*Li*_(*t*)] on [0,*T*] (Legendre polynomials or Fourier basis are implemented in the software, however any other bases can be theoretically considered) of gene specific degree 0 ≤ *L_i_* ≤ *L*_max_ with coefficients $C_{i}^{\left(l\right)},\;l\;=\;0,\;\cdots \;,\;L_{i}$:

$$\mu_{i}\left(t\right)\;=\sum_{l=0}^{L_{i}}c_{i}^{\left(l\right)}\phi_{l}\;\left(t\right).$$

Similar to EDGE, the objective is to identify the genes showing different functional expressions between treatment and control (i.e. μ*_i_*(*t*) ≠ 0), and additionally to explicitly evaluate the effect of the treatment (i.e., estimate ${\hat{\mu }}_{i}\left(t\right)$), which in EDGE is hidden in the model but it could be obtained by least squares fit of (1) under model (2). Following Angelini *et al*. [18], genes are treated as conditionally independent and modeled as

$$z_{i}=D_{i}c_{i}+\;\zeta_{i}$$

in which **D**_*i*_ is the block design matrix, the *j*-row of which is the block vector [φ_0_(*t_j_*) φ_1_(*t_j_*) ··· φ_*Li*_(*t_j_*)] replicated $k_{j}^{i}$ times; $z_{i}=\;{\left(z_{i}^{1,1}\;...\;z_{i}^{1,k_{1}},\;\cdots ,z_{i}^{n,1},\;...\;z_{i}^{n,k_{n}}\right)}^{T},\;c_{i}\;=\;{\left(c_{i}^{0},\;...,c_{i}^{L_{i}}\right)}^{T}$ and $\zeta_{i}\;=\;{\left(\zeta_{i}^{1,1},\;...,\;\zeta_{i}^{1,k_{1}},\;\cdots ,\zeta_{i}^{n,1},\;...,\;\zeta_{i}^{n,k_{n}}\right)}^{T}$ are, respectively, the column vectors of all measurements for gene *i*, the coefficients of μ*_i_*(*t*) in the chosen basis, and random errors. The following hierarchical model is imposed on the data:

$$\begin{array}{r} z_{i}|L_{i},c_{i},\sigma^{2}\\ L_{i}\\ c_{i}|L_{i},\sigma^{2} \end{array}\;\;\;\;\;\;\;\;\;\;\;\;\;\;\begin{array}{c} ∼\\ ∼\\ ∼ \end{array}\;\;\;\;\;\;\;\;\;\;\;\;\;\;\;\begin{array}{l} N\left(D_{i}c_{i},\sigma^{2}I_{M_{i}}\right)\\ \text{Truncated Poisson }\left(\lambda ,L_{\max}\right)\\ \pi_{0}\delta \left(0,\ldots ,0\right)+\left(1-\pi_{0}\right)N\left(0,\sigma^{2}\tau_{i}^{2}Q_{i}^{-1}\right) \end{array}$$

All parameters in the model are treated either as random variables or as nuisance parameters that are recovered from data. Noise variance σ^2^ is assumed to be random, σ^2^ ˜ ρ(σ^2^) in order to account for possibly non-Gaussian errors which are quite common in microarray experiments.

Three different Bayesian models are contained in BATS providing the user a more flexible theoretical set-up to accommodate various types of error distributions, namely, all scale mixtures of a normal distribution: delta-type prior $\rho \left(\sigma^{2}\right)=\delta \left(\sigma^{2}-\sigma_{0}^{2}\right)$, the inverse Gamma prior ρ(σ^2^) = *IG*(γ, *b*) and the exponential type prior $\rho \left(\sigma^{2}\right)\;=\;c_{\mu }\sigma^{M_{i}-1}e^{-\sigma^{2}\mu /2}$ which lead to normal, Student *T* and double-exponential errors, respectively. The choice of differentially expressed genes is made on the basis of Bayes Factors which are used for multiplicity control and are computed using the procedure described by Abramovich *et al*. [34]. Once significant genes are detected, the coefficients ${\hat{c}}_{i}^{\left(l\right)}$ and, subsequently, the curve ${\hat{\mu }}_{i}\left(t\right)$ are estimated by the posterior means. Hyperparameters π_0_ and $\sigma_{0}^{2}$, γ, *b* or μ are estimated from the data, or can be entered as known by the user. Gene specific parameters $\tau_{i}^{2}$ and *L_i_* are estimated by maximizing the marginal likelihood *P(z_i_)* and the posterior mean or mode of *P*(*L_i_*|**Z**_*i*_), respectively.

The advantage of the Bayesian model described above is that since all priors are conjugate (see [18] for details), all posterior inference can be carried out analytically with very efficient computations.

The method is used for simultaneous estimation of the curves, as well as for ranking the curves (genes) according to their significance level. Moreover, significance testing of the curves is carried out by controlling the multiplicity of comparisons from a Bayesian perspective [34], providing an automatic cut-off. We performed the analysis by using two error models (the normal and the double-exponential) and a range of values of the parameter λ, which influences the prior degree of the polynomial curve estimated for each gene.

### Simulations

To compare performances of EDGE, *timecourse* and BATS, we carried out a small simulation study by generating data with the Simulation utility of BATS. We generated data to mimic the structure of the real data set described above, with *N* = 10000, *n* = 11 and $k_{i}^{j}=2$ for all *j* = 1, …, 11 except $k_{i}^{4}\;=\;3$. In the data sets generated, 1000 or 2000 genes were randomly chosen to be “differentially expressed”, corresponding respectively to 10 % or 20 % of the total number of genes. The first scenario correspond to a case where relatively few genes are involved in the process, the second to a more strong respondence to the treatment. The values of 1000 and 2000 where chosen from the prior belief on behavior of the real data experiments. The remaining 9000 or 8000 curves were set to identical zero.

For each significant curve, the Simulation utility samples the degree of the polynomial $L_{i}^{true}$ from a discrete uniform distribution in [1, *L*_max_], with *L*_max_ = 6 (in contrast to the truncated Poisson that is used in fitting the model). Polynomials of degree zero are excluded since a nonzero constant signal is questionable from a biological point of view. Coefficients c_*i*_ where randomly sampled from $N\left(0,\;\sigma^{2}\tau_{i}^{2}Q_{i}^{-1}\right)$. Matrix **Q**_*i*_ is set to $Q_{i}\;=\;\text{diag}\left(1^{2\nu_{i}},\;2^{2\nu_{i}},\;...,\;L_{i}^{2\nu_{i}}\right)$ where ν_*i*_ ∼ *U*([0,1]) and $\tau_{i}^{2}$ was sampled uniformly in order to produce the signal-to-noise ratio (SNR) in the interval between 2 and 6. Under this set up we can mimic both weak and strong signals and different signal regularity (which is not accounted explicitly by any of the models). Furthermore, since is known that noise on microarray date has heavier tails than gaussian, we performed simulations under three scenarios of i.i.d. noise: normal *N*(0, σ^2^) and Student *T* with 5 or 3 degrees of freedom (indicated as *T*5 and *T*3, respectively). Student noise was rescaled to have the same variance σ^2^ of the normal case (σ = 0.33, the estimated value for the real data set).

In addition, very large values (with a threshold of 5) were filtered out and substituted with missing values, mimicking real data preprocessing where unreliable values are eliminated.

For each kind of noise and number of true signals we generated 5 data-set, averaging the results. Analysis of simulated data was performed with the three methods with the same choice of parameters used in the real data analysis: EDGE *q*-value 0.01 and 0.001; BATS error model normal and double exponential and λ = 9 and λ = 12; with *timecourse* we chose the first genes in the ranked list corresponding to the same number of the genes selected by BATS on the same dataset, to evaluate the number of false positives and false negatives.

### Cluster analysis

Cluster analysis on the final list of gene profiles significantly affected by estrogen stimulation was performed using a Bayesian functional based software, Splinecluster [35]. The method proposes a hierarchical cluster approach, where the number of cluster is automatically selected by maximizing the marginal distribution. However, it is recommended both for computational and for practical point of view to apply the method only on the relevant subset of genes, instead of the whole dataset of genes. Here, similarly to BATS, the gene profiles are also represented by expansions over a certain basis and the normal-inverse gamma prior is imposed on the unknown coefficients. The number of clusters and cluster participation are also treated as random, leading to a full Bayesian model. Since the method does not address many of the issues which we treat in the Results and discussion Section, we processed the selected data matrix by filtering out missing data points and by averaging the replicates at each time point.

## Results and discussion

### Experimental design of the experiment and its implications

We present the analysis performed on a time series of microarray data from breast cancer cells treated with estrogens. Our experimental design is formalized in a ‘one sample’ statistical model with a time series, in which replicated arrays for each time-point are technical replicates, with no special relationships between each other. We also have unequally spaced sampling intervals (1h between the first two time-points, 2h till the time-point of 8h and 4h till the end of the series) and 2 replicates at each time-point, except one case (4h) in which we have 3 replicates. This data structure has quite common features in microarray experimental designs: a number of replicates barely sufficient to get statistically significant results, unequal number of replicates which may be due to technical needs or reasons of biological interest. For example, the higher detail in the first part of the curve reflects a greater interest from a biological point of view in the earlier responses to hormone treatment with respect to the rest of the time series. Some difficulties may arise in analyzing data presenting features like these, both for a static analysis approach and with a longitudinal method. In fact, for a static method of comparison treated/non treated, performed point-by-point, the number of replicates of individual time-points is lower than the required minimum of most standard tests. This limited number of replicates is justified by the time-series analysis: since we are interested in the whole profile, we don't need absolute precision in each time point comparison but rather we need to take advantage of the temporal structure of the data and use all information available along the time in order to make appropriate and robust inference.

### Pre-processing

We evaluated the effect of different normalization algorithms in terms of overlap between the selected gene lists produced with the time-series analysis methods used. After inspection of normalized data, the cubic spline method was discarded since the data produced was not correctly normalized between the arrays (see Additional file 1), thus requiring further manipulation on data that we decided not to apply. The better overlap was noted between quantile and lumi normalization, with average being the best performing algorithm among the ones present in BeadStudio software.

After the filtering step, the genes left for the analysis were 9593, of which 1261 (13.2%) had between 1 and 4 missing values.

### Time series analysis

#### Sliding window analysis

This method is quite naive and is presented just to have a static counterpart to compare with the other methods. We chose to apply it only to data normalized with BeadStudio algorithms, thus representing an analysis performed with the only help of the chip manufacturer's software. We applied the internal differential analysis algorithm which uses the bead standard deviation in the error model, thus making it possible to analyze data with only 2 replicates for each time-point, as in our case, unlike a standard t-test. Results of the analysis with this and the other methods are reported in Table 1. We noted a fairly good robustness to normalization effects (75-80% overlap among the selected gene lists). Although being a very simple procedure, we obtained results which were comparable to other methods having more appropriate assumptions (60-70% with EDGE and BATS). However, we also have to point out that, by considering a window of three time points regardless of time interval between them, we are incorrectly treating unequally spaced times with the same weight in the analysis. It can nevertheless be useful to detect local changes in the expression.

**Table 1:** **Comparison of the selected gene lists obtained with different methods of selection**. Numbers indicate the genes obtained by pairwise intersection of different methods of selection. In bold are the selected gene lists for each method.

	Sliding window 20^1^	Sliding window 30^1^	EDGE 0.01^2^	EDGE 0.001^2^	BATS #1^3^	BATS #2^4^	*timecourse* 1000^5^	*timecourse* 1500^5^
Sliding window 20^1^	**1563**	997	1126	667	903	1069	140	209
Sliding window 30^1^		**997**	825	540	690	797	85	128
EDGE 0.01^2^			**2595**	1145	936	1086	232	343
EDGE 0.001^2^				**1145**	590	659	104	154
BATS #1^3^					**1478**	1397	157	157
BATS #2^4^						**1660**	232	243
*timecourse* 1000^5^							**1000**	1000
*timecourse* 1500^5^								**1500**

^1^DiffScore threshold. ^2^q-value threshold. ^3^Error model = normal, λ=12. ^4^Error model = double-exponential, λ=9. ^5^Number of ranked genes selected.

#### EDGE

EDGE is distributed as a stand-alone software and, although relying on R [21], it silently uses it in the background, so that the user does not need to know the language to use it but only interacts with a graphical interface. It also has some useful utilities to inspect the input data, such as the possibility to make boxplots, to check the presence of missing data and to impute them with the KNN algorithm. The results are highly robust to changing normalizations (80-96% overlap among all the four methods) except for the case of rank invariant normalization, with which the number of significant genes drops unexpectedly with respect to the others. We obtained similar results both by filtering out missing data and by imputing them. As compared with the other methods, on real data EDGE selects a surprisingly much longer list of genes (Table 1). Moreover, we observed that, even though we increased the number of permutations, due to the granularity problem, genes with the same *q*-value are too many, since for example the first 67 (average norm.), 44 (lumi) or 85 (quantiles) genes all result as ‘first rank’ genes with the same q-value. To reduce granularity one should further increase the number of permutations, but then as a consequence the computational cost would also increase, thus making the method less convenient to use.

#### timecourse

*timecourse *[32] is a package distributed with Bioconductor [17], thus requiring knowledge of the statistical environment R [21], which is both an advantage for those familiar with this language, since it is very quick to install and use new packages, but it can be unfriendly for biologists. Similarly to EDGE, we found very similar results both when filtering out genes with missing observations and when imputing them. This method only ranks in order of significance the input gene list without providing an automatic or suggested cut-off to determine which genes are significant. For this reason, on real data we selected the first 1000 and 1500 genes of the rank ordered lists to compare results among normalizations and with the other methods. Surprisingly, we found a very low overlap both between the ordered lists prepared with different normalizations and with lists produced with other methods (Table 1). It is worth mentioning that our dataset contains only technical (indistinguishable) replicates, thus the method could not take advantage of the replicate identification, nonetheless the difference with the other methods and above all between data normalized with different methods is difficult to explain.

#### BATS

**Figure 1:**
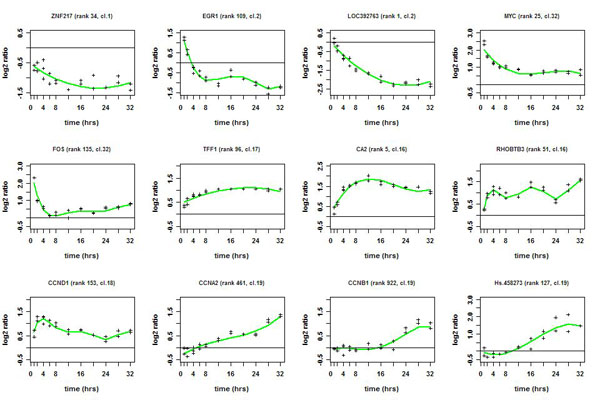
**Expression kinetics of representative estrogen-responsive genes.** Green lines represent the estimated profiles generated by BATS for each gene and crosses show the actual data of replicates.

BATS is also distributed as a stand-alone software with a graphical and friendly interface, as, although written in Matlab [36] it does not require the use of Matlab. Selection was found robust with respect to changes in parameters (85-90% genes common to all the combinations used) and type of normalization (74-82%, with a lower overlap for the rank invariant). BATS has also some graphical utilities to plot, filter data and compare resulting lists and is the only method which allows to save the estimated profile for the selected genes for further use (Figure 1). As the result on the ‘one sample’ problem, the technique allows different number of basis functions for each curve, which improves the fits, it does not require to pre-determine the most significant genes to select the dimension of the fit and avoids a computer intensive evaluation of the *p*-values via bootstrap. Furthermore, by using the Bayesian formulation in combination with the functional approach it can successfully handle various technical difficulties which arise in microarray time-course experiments such as a small number of observations available, non-uniform sampling intervals, presence of missing data or multiple data as well as temporal dependence between observations for each gene, which are not completely addressed by the above mentioned methods. On the other hand, current version of the BATS method cannot be applied to the ‘two sample’ case.

### Comparison of methods

#### Simulation study

Tables 2 and 3 summarize results with the simulated datasets. In particular, for any group of datasets it is reported the average number of rejected hypotheses, i.e. genes declared differentially expressed, the average number of the correctly rejected hypotheses, the false discovery rate, estimated as the average proportion of the falsely rejected hypotheses over the total number of rejected hypotheses, and the false negative rate, estimated as the average proportion of the significant curves not detected over the number of not rejected hypothesis. As already stated, since *timecourse* does not provide any cut-off point, for the sake of comparison we cut the ranked list on the same number of significant genes as in BATS with default parameters choice. We can say that all methods have good performances under all the simulated datasets, with BATS providing more accurate results (both in terms of FDR and FNR) than the other methods. However, we have to note that the simulated datasets were generated according to several of the BATS model assumptions. On the other hand it does not exists an accepted standard dataset of microarray time course to be used as benchmark, neither a way to perform a blind experiment, or a well established set of synthetic test functions as in non parametric regression. Different methods account for different biological information and are valid under different assumptions, while the various amount of different interactions and sources of error that can affect the data can often change the performance of a given method from a simulated case to the real data application.

**Table 2:** **Simulation study**. Datasets generated with 1000 true signals, with three different noise models. Results were averaged over 5 datasets.

	*Noise model N*				*Noise model T5*				*Noise model T3*			
Method	Rej.^4^	Corr.^5^	FDR^6^	FNR^7^	Rej.^4^	Corr.^5^	FDR^6^	FNR^7^	Rej.^4^	Corr.^5^	FDR^6^	FNR^7^
EDGE^1^ 0.01	383.8	382.4	0.004	0.064	405.6	403.4	0.005	0.062	462.6	460.8	0.004	0.057
EDGE^1^ 0.001	207.8	207.4	0.002	0.081	183.6	183.6	0.000	0.083	187	187	0.000	0.083
*timecourse*^2^	775.6	733.4	0.054	0.029	794.4	690.6	0.131	0.034	869.8	733.4	0.157	0.029
BATS^3^ N, 9	775.6	775.6	0.000	0.024	794.4	782.6	0.015	0.024	869.8	803.4	0.076	0.022
BATS^3^ N, 12	775.4	775.4	0.000	0.024	794.2	782.4	0.015	0.024	869	802.6	0.076	0.022
BATS^3^ D, 9	753.2	753.2	0.000	0.027	774.8	762.8	0.015	0.026	875.4	793.4	0.094	0.023
BATS^3^ D, 12	745.8	745.8	0.000	0.027	768.4	756.4	0.016	0.026	871.6	789.2	0.095	0.023

^1^q-value threshold.

^2^Number of rejected chosen equal to the case of BATS (N,9), for comparison purpose.

^3^Error model N = normal, D = double-exponential, the indicated number is the value of λ.

^4^Rej. (Rejected) = average number of genes declared differentially expressed.

^5^Corr. (Correct) = average number of the correctly rejected hypotheses.

^6^FDR (False Discovery Rate) = average proportion of falsely rejected hypotheses over the total number of rejected hypotheses.

^7^FNR (False Negative Rate) = average proportion of false negatives over the total number of not rejected hypotheses.

**Table 3:** **Simulation study**. Datasets generated with 2000 true signals, with three different noise models. Results were averaged over 5 datasets.

	*Noise model N*				*Noise model T5*				*Noise model T3*			
Method	Rej.^4^	Corr.^5^	FDR^6^	FNR^7^	Rej.^4^	Corr.^5^	FDR^6^	FNR^7^	Rej.^4^	Corr.^5^	FDR^6^	FNR^7^
EDGE^1^ 0.01	928.4	921.8	0.007	0.119	953.4	948	0.006	0.1163	1054.6	1048.2	0.006	0.106
EDGE^1^ 0.001	519.6	519.6	0.000	0.156	526	526	0.000	0.1556	544.6	544.6	0.000	0.154
*timecourse*^2^	1386	1380	0.004	0.072	1396	1319	0.055	0.0791	1461	1384	0.052	0.072
BATS^3^ N, 9	1385.8	1385.8	0.000	0.071	1395.8	1391	0.003	0.0708	1460.6	1435	0.018	0.066
BATS^3^ N, 12	1382	1382	0.000	0.072	1393.4	1388.6	0.003	0.0710	1459.6	1433	0.018	0.066
BATS^3^ D, 9	1386	1386	0.000	0.071	1407.2	1403.4	0.003	0.0694	1510.2	1477.4	0.022	0.062
BATS^3^ D, 12	1368.2	1368.2	0.000	0.073	1384.2	1380.4	0.003	0.0719	1489.8	1457	0.022	0.064

^1^q-value threshold.

^2^Number of rejected chosen equal to the case of BATS (N,9), for comparison purpose.

^3^Error model N = normal, D = double-exponential, the indicated number is the value of λ.

^4^Rej. (Rejected) = average number of genes declared differentially expressed.

^5^Corr. (Correct) = average number of the correctly rejected hypotheses.

^6^FDR (False Discovery Rate) = average proportion of falsely rejected hypotheses over the total number of rejected hypotheses.

^7^FNR (False Negative Rate) = average proportion of false negatives over the total number of not rejected hypotheses.

For what concerns EDGE, we observe a quite conservative behavior (it has a higher FNR with respect the other methods) which is not preserved on the analysis of real data. This might be due to the bootstrap technique applied to estimate the parameters.

In the case of *timecourse*, we note a higher consistency with the other methods, in spite of its strikingly different results when applied on real data. It is not surprising to observe that the methods performed differently on real data with respect to simulated data, since any simulation has implicit assumptions which may or may not be verified on experimental datasets. Apparently, a more irregular noise distribution on real data has arisen opposite problems to EDGE and *timecourse* in detecting gene expression signals over the noise, while on the contrary it does not affect the performance of BATS significantly.

#### Real data analysis

When several methods are compared on experimental data, there is no clear and well accepted way to compare performance of each approach and the final choice usually depends upon several considerations. We thus first investigated the robustness of each procedure in terms of user selected parameters and different normalization procedures (data not shown). In Table 1 are reported the results relative to the gene lists selected by each of the procedures described above, all normalized according to the average method. As shown, the less rigorous sliding window approach as well as EDGE and BATS have a satisfying overlap among the gene list they select. We then considered the different methods from a statistical point of view, analyzing benefits and drawbacks.

Sliding windows is of course the less statistically rigorous, it does not take into account unequally spaced time points or missing data nor provides a global measure of significance for the whole time series. On the other hand, this is very intuitive and computationally inexpensive, and may be useful to detect local changes.

EDGE, on the other hand, suffers for the problem of the granularity of *p*-values which can be only partially solved by increasing the number of iterations, although at the price of a high computational cost, which can become prohibitive for large dataset. Moreover, the choice of an appropriate threshold may become problematic, since small changes lead to remarkable differences in the selected gene lists. Furthermore, EDGE assumes the same degree in the functional expansion of each gene and, as a consequence, it may lack in adaptation. It has, however, the merit of being the first tool to formalize the problem of selection by a functional approach.

*timecourse* is mainly designed for a slightly different problem, hence its use in the context considered here does not allow to take complete advantage of the methods itself. Moreover, similarly to EDGE, *timecourse* does not account for missing data, requiring the user to filter out incomplete datasets, missing time points, or to force the user to employ preliminary procedures in order to impute them. Furthermore, this method does not provide an automatic cut-off for selecting significant genes, nor uses the quantitative ‘time’ information in an explicit way.

Hence, we found BATS more appropriate for this experimental setting, since it automatically accounts for various technical difficulties which arise in microarray time course experiments, such as limited number of observations, non uniform sampling intervals or missing/multiple records, all conditions which are not completely addressed by the above mentioned alternative methods. Moreover, since BATS does not require bootstrap and posterior inference can be evaluated in closed form, and it is applicable also to the larger datasets that are becoming more widely used due to microarray technology improvements and diffusion. Furthermore, it has the merit of providing an estimate of the significant expression profile, which is not explicitly provided by any of the other methods, while being also very flexible, capable of handling gene specific variance and, using the Bayesian paradigm, allowing better adaptation of the estimates to the underlying data.

### Cluster analysis

**Figure 2:**
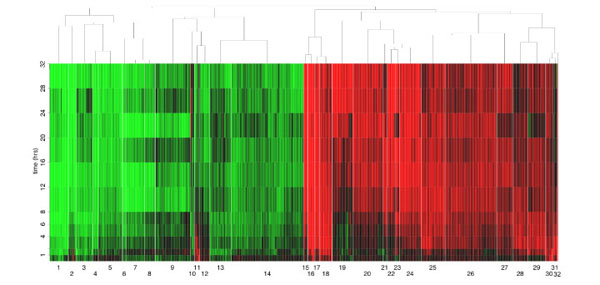
**Heatmap of co-regulated gene clusters.** Hierarchical representation of the 32 clusters generated by Splinecluster.

**Figure 3:**
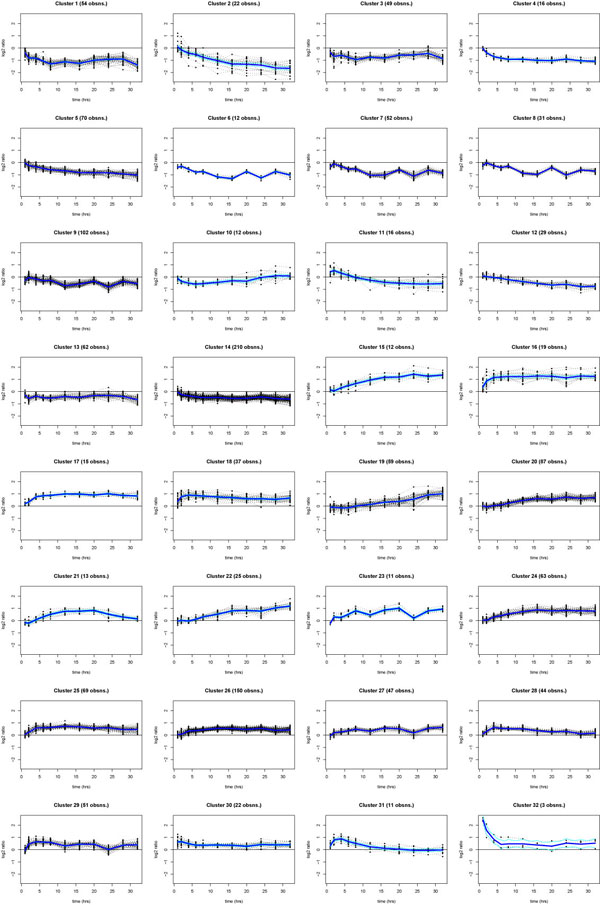
**Cluster profiles**. Blue lines show each cluster average profile.

The biological model selected for this study is based on the responsiveness of human breast cancer ZR-75.1 cells to stimulation with estrogen, since it is well known that under these conditions the hormone evokes in the cell complex, timed gene regulation events that result in cell cycle progression and inhibition of cell death [3,4] and changes in cell metabolism and function [2,5]. This is accomplished by hormonal activation of different signal transduction cascades leading, among other, to physical and functional interactions of activated ERs with the genome [1,37]. Correct identification of gene clusters that shows synchronous responses to estrogen is thus a key step to dissect the molecular mechanisms that underlie cell regulation by these steroid hormones. We chose the Splinecluster method [35] to identify homogeneous time clusters within the final list of estrogen regulated genes selected since we wanted to use a clustering approach which also would take into account the temporal relationship among samples, as a natural subsequent choice. Considering the amount of noise which usually affects microarray experiments and the dimensionality of the problem, we stress that in order to reduce the computational complexity of any clustering procedure, while obtaining more significant results, it is of great importance to perform in any case the analyses only on data relative to the subset of genes which do respond to the treatment. In Figures 2 and 3 are displayed the results of cluster analyses carried out on the set of estrogen-regulated genes from ZR-75.1 cells selected with BATS according to the following parameter settings: normal error model and λ = 12. The actual data are provided in Additional file 2, which includes also the final gene list.

## Conclusions

Microarray experiments enable to study at genome-wide level the dynamics of gene regulation events. Since thousands of genes are spotted in modern platforms, the amount of data provided is relevant, hence it is important to have an automatic, statistically robust, computationally fast and flexible procedure to select gene expression profiles which show significant changes in time.

We tested different methods tailored for analyzing data derived from time-course microarray experiments, which can be modeled under a ‘one sample’ framework, in order to find the most appropriate analysis pipeline to use in future experiments.

We evaluated advantages and limits of each method assessed, in terms of usability, computational burden, flexibility to characteristics of microarray experimental designs, robustness to normalizations and overlap with the other methods. We have found an analysis pipeline of R/Bioconductor preprocessing and then selection of significant genes with BATS to be the most appropriate in the ‘one sample’ case. Selected genes can then be clustered with Splinecluster [35], which is a method which uses a functional approach, coherently with the selection procedure used.

To validate the biological significance of the gene expression profiles and gene clusters here identified, these were compared with the results we obtained previously in estrogen-stimulated ZR-75.1 cells under identical experimental conditions [4]. The majority of the genes in common among the two lists showed very similar/identical pattern of expression. This is evident, for example, when comparing in Additional file 2 and Cicatiello *et al*. [4] the data relative to: *EGR1* (early growth response 1: cluster 1), *ZNF217* (zinc finger protein 217: cluster 1), *MYC* (c-myc: cluster 32), *FOS* (c-fos: cluster 32), *TFF1* (trefoil factor 1: cluster 17), *CCND1* (cyclin D1: cluster 18), *CCNA2* (cyclin A2: cluster 19) and *CCNB1* (cyclin B1: cluster 19). All these genes are known target of ERs, while their activity relates to regulation of cell cycle phasing. The pattern of induction/repression of these genes by estrogen over time (see also Figure 1), as identified with the method here described, perfectly corresponds to their known biological role in these cells, providing a strong biological confirmation of the reliability of the gene selection method proposed.

## Competing interests

The authors declare that they have no competing interests.

## Authors' contributions

MM participated in the conception and design of the study, performed the statistical analyses and participated in drafting the manuscript. LC, LF and MR carried out the microarray experiments and participated in data analyses. OMVG contributed to the statistical analyses and to manuscript drafting. AMF and CA participated in the conception and design of the study and supervised the analyses. AW coordinated the project, participated in conception and design of the study and participated in drafting and finalization of the manuscript. All authors read and approved the final manuscript.

## Supplementary Material

Additional file 1: Normalization boxplots Boxplots of normalized data after filtering and log-transformation. Click here for file

Additional file 2: Selected gene list Selected gene list corresponding to the cluster analysis shown in Figure 2 and 3. Each gene is accompanied by its measured signal over time (data shows average *log*_2_ ratios, normalized with quantile method). Genes with one or more missing points are listed at the bottom, since the clustering software could not include them in the analysis. Click here for file

## References

[B1] Weisz A, Oettel M, Schillinger E (1999). Estrogen regulated genes. Handbook of Experimental Pharmacology, Volume 135/I: Estrogens and Antiestrogens.

[B2] Weisz A (2005). New insights on estrogen action from gene expression profiling. Signal Transduction and Neoplastic Transformation in Endocrine Systems: Molecular mechanisms and clinical aspects, Volume 211.

[B3] Weisz A, Addeo R, Altucci L, Battista T, Boccia V, Cancemi M, Cicatiello L, Germano D, Mancini A, Pacilio C, Bresciani F, Edited by Kuramoto H, Gurpide E, Tokyo (1996). Molecular mechanisms for estrogen control of cell cycle progression during G1. Sex Steroid Hormone Action.

[B4] Cicatiello L, Scafoglio C, Altucci L, Cancemi M, Natoli G, Facchiano A, Iazzetti G, Calogero R, Biglia N, De Bortoli M, Sfiligol C, Sismondi P, Bresciani F, Weisz A (2004). A genomic view of estrogen actions in human breast cancer cells by expression profiling of the hormone-responsive transcriptome. J Mol Endocrinol.

[B5] Scafoglio C, Ambrosino C, Cicatiello L, Altucci L, Ardovino M, Bontempo P, Medici N, Molinari AM, Nebbioso A, Facchiano A, Calogero R, Elkon R, Menini N, Ponzone R, Biglia N, Sismondi P, De Bortoli M, Weisz A (2006). Comparative gene expression profiling reveals partially overlapping but distinct genomic actions of different antiestrogens in human breast cancer cells. J Cell Biochem.

[B6] Tusher V, Tibshirani R, Chu C (2001). Significance analysis of microarrays applied to the ionizing radiation response. Proc Natl Acad Sci U S A.

[B7] Kerr MK, Martin M, Churchill GA (2000). Analysis of variance for gene expression microarray data. J Comput Biol.

[B8] Park T, Yi SG, Lee S, Lee SY, Yoo DH, Ahn JI, Lee YS (2003). Statistical tests for identifying differentially expressed genes in time course microarray experiments. Bioinformatics.

[B9] Conesa A, Nueda MJ, Ferrer A, Talon M (2006). MaSigPro: a method to identify significantly differential expression profiles in time-course microarray-experiments. Bioinformatics.

[B10] Di Camillo B, Sanchez-Cabo F, Toffolo G, Nair SK, Trajanosky Z, Cobelli C (2005). A quantization method based on threshold optimization for microarray short time series. BMC Bioinformatics.

[B11] Smyth GK, Gentleman R, Carey V, Dudoit S, Irizarry R, Huber W, Springer (2005). Limma: linear models for microarray data. Bioinformatics and Computational Biology Solutions using R and Bioconductor.

[B12] De Hoon MJL, Imoto S, Miyano S (2002). Statistical analysis of a small set of time-ordered gene expression data using linear splines. Bioinformatics.

[B13] Bar-Joseph Z (2004). Analyzing time series gene expression data. Bioinformatics.

[B14] Storey JD, Xiao W, Leek JT, Tompkins RG, Davis RW (2005). Significance analysis of time course microarray experiments. Proc Natl Acad Sci U S A.

[B15] Leek J, Monsen E, Dabney A, Storey J (2006). EDGE: extraction and analysis of differential gene expression. Bioinformatics.

[B16] Tai YC, Speed TP (2006). A multivariate empirical Bayes statistic for replicated microarray time course data. Ann Statist.

[B17] Gentleman R, Carey V, Bates D, Bolstad B, Dettling M, Dudoit S, Ellis B, Gautier L, Ge Y, Gentry J, Hornik K, Hothorn T, Huber W, Iacus S, Irizarry R, Leisch F, Li C, Maechler M, Rossini A, Sawitzki G, Smith C, Smyth G, Tierney L, Yang JYH, Zhang J (2004). Bioconductor: open software development for computational biology and bioinformatics. Genome Biol.

[B18] Angelini C, De Canditiis D, Mutarelli M, Pensky M (2007). A Bayesian Approach to Estimation and Testing in Time-course Microarray Experiments. Stat Appl Genet Mol Biol.

[B19] Angelini C, Cutillo L, De Canditiis D, Mutarelli M, Pensky M (2007). BATS: a Bayesian user-friendly software for Analyzing Time Series microarray experiments. Rapp Tech IAC-CNR 331-07.

[B20] Gunderson KL, Kruglyak S, Graige MS, Garcia F, Kermani BG, Zhao C, Che D, Dickinson T, Wickham E, Bierle J, Doucet D, Milewski M, Yang R, Siegmund C, Haas J, Zhou L, Oliphant A, Fan J, Barnard S, Chee MS (2004). Decoding randomly ordered DNA arrays. Genome Res.

[B21] Everitt B, Hothorn T A Handbook of Statistical Analyses Using R. In Genome Res.

[B22] (2005). Illumina Inc. BeadStudio User Guide.

[B23] Workman C, Jensen L, Jarmer H, Berka R, Gautier L, Nielser H, Saxild H, Nielsen C, Brunak S, Knudsen S (2002). A new non-linear normalization method for reducing variability in DNA microarray experiments. Genome Biol.

[B24] Bolstad BM, Irizarry RA, Astrand M, Speed TP (2003). A comparison of normalization methods for high density oligonucleotide array data based on bias and variance. Bioinformatics.

[B25] Barnes M, Freudenberg J, Thompson S, Aronow B, Pavlidis P (2005). Experimental comparison and cross-validation of the Affymetrix and Illumina gene expression analysis platforms. Nucleic Acids Res.

[B26] Gautier L, Cope L, Bolstad BM, Irizarry RA (2004). affy-analysis of Affymetrix GeneChip data at the probe level. Bioinformatics.

[B27] Du P, Kibbe W, Lin S (2007). Using lumi, a package processing Illumina Microarray. http://www.bioconductor.org.

[B28] Huber W, von Heydebreck A, Sultmann H, Poustka A, Vingron M (2002). Variance Stabilization Applied to Microarray Data Calibration and to the Quantification of Differential Expression. Bioinformatics.

[B29] Efron B, Tibshirani R (1993). An Introduction to the Bootstrap.

[B30] Storey J, Tibshirani R (2003). Statistical significance for genomewide studies. Proc Natl Acad Sci U S A.

[B31] Troyanskaya O, Cantor M, Sherlock G, Brown P, Hastie T, Tibshirani R, Botstein D, Altman RB (2001). Missing value estimation methods for DNA microarrays. Bioinformatics.

[B32] Tai Y, Speed T, Edited by Nuber U (2005). Statistical analysis of microarray time course data. DNA Microarrays.

[B33] Hastie T, Tibshirani R, Sherlock G, Eisen M, Brown P, Botstein D (1999). Imputing Missing Data for Gene Expression Arrays. Stanford University Statistics Department Technical report.

[B34] Abramovich F, Angelini C (2006). Bayesian Maximum a Posteriori Multiple Testing Procedure. Sankhya - The Indian Journal of Statistics.

[B35] Heard N, Holmes C, Stephen D (2006). A quantitative study of gene regulation involved in the Immune response of Anopheline Mosquitoes: An application of Bayesian hierarchical clustering of curves. J Amer Stat Soc.

[B36] The MathWorks I. Getting Started with MATLAB 7.

[B37] Cicatiello L, Addeo R, Sasso AR, Altucci L, Belsito Petrizzi V, Borgo R, Cancemi M, Caporali S, Caristi S, Scafoglio C, Teti D, Bresciani F, Perillo B, Weisz A (2004). Estrogens promote persistent G1 activation of the CCND1 gene by inducing transcriptional de-repression via c-Jun/c-Fos/ER complex assembly to a distal regulatory element and recruitment of cyclin D1 to its own gene promoter. Mol Cell Biol.

