# Supplementary figures and images for: Time-course analysis of genome-wide gene expression data from hormone-responsive human breast cancer cells
Source: BMC Bioinformatics. 2008 Mar 26;9(Suppl 2):S12. doi: 10.1186/1471-2105-9-S2-S12 (PMC2323661; doi:10.1186/1471-2105-9-S2-S12)

average

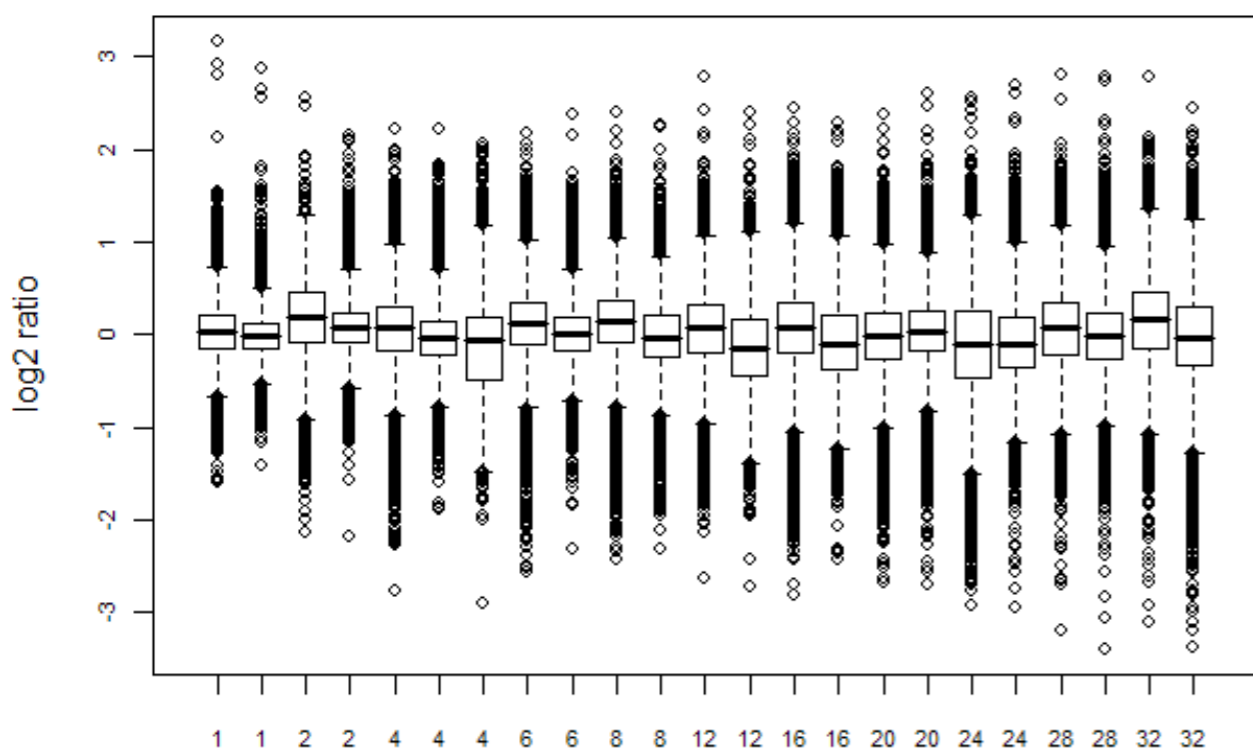

rank invariant

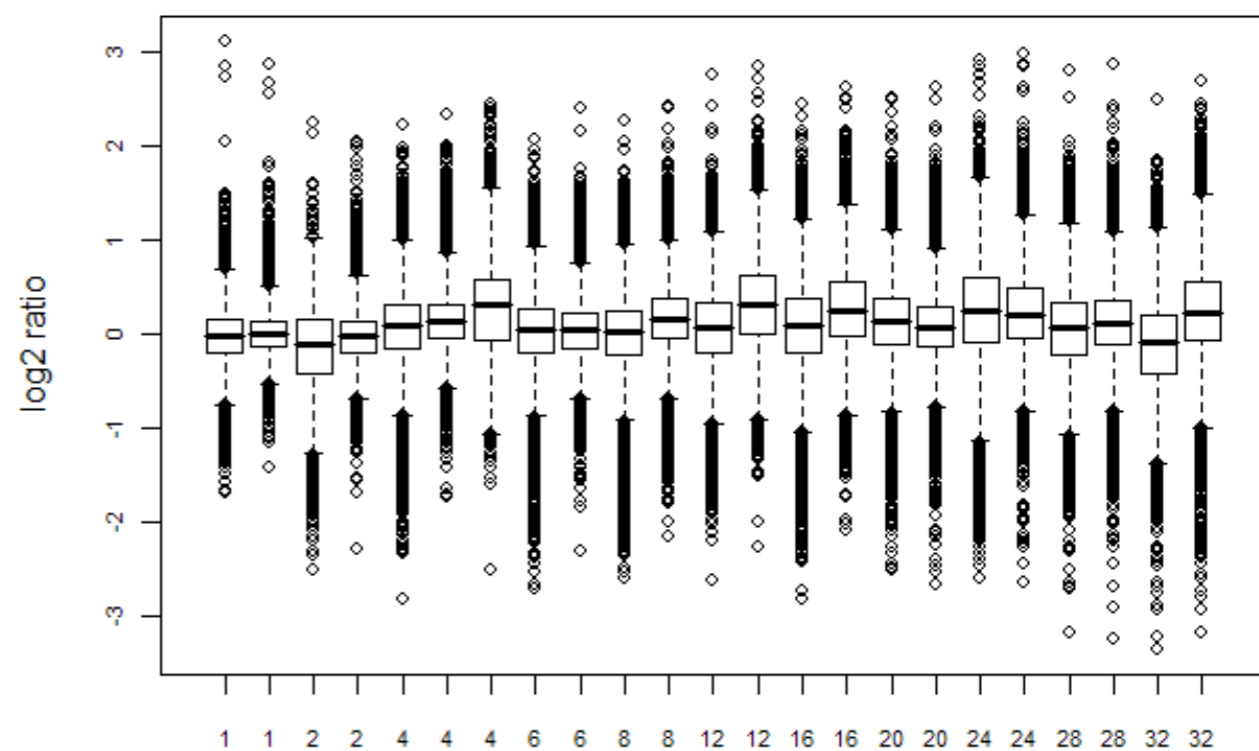

cubic spline

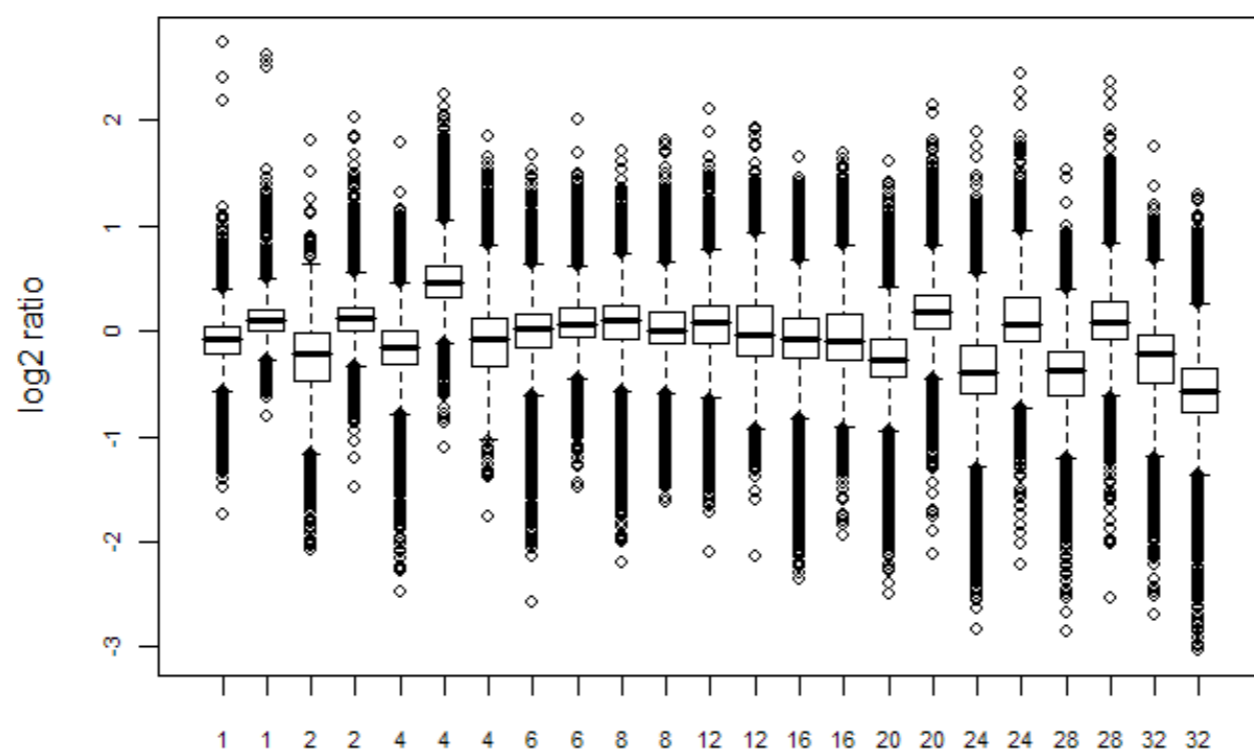

quantile

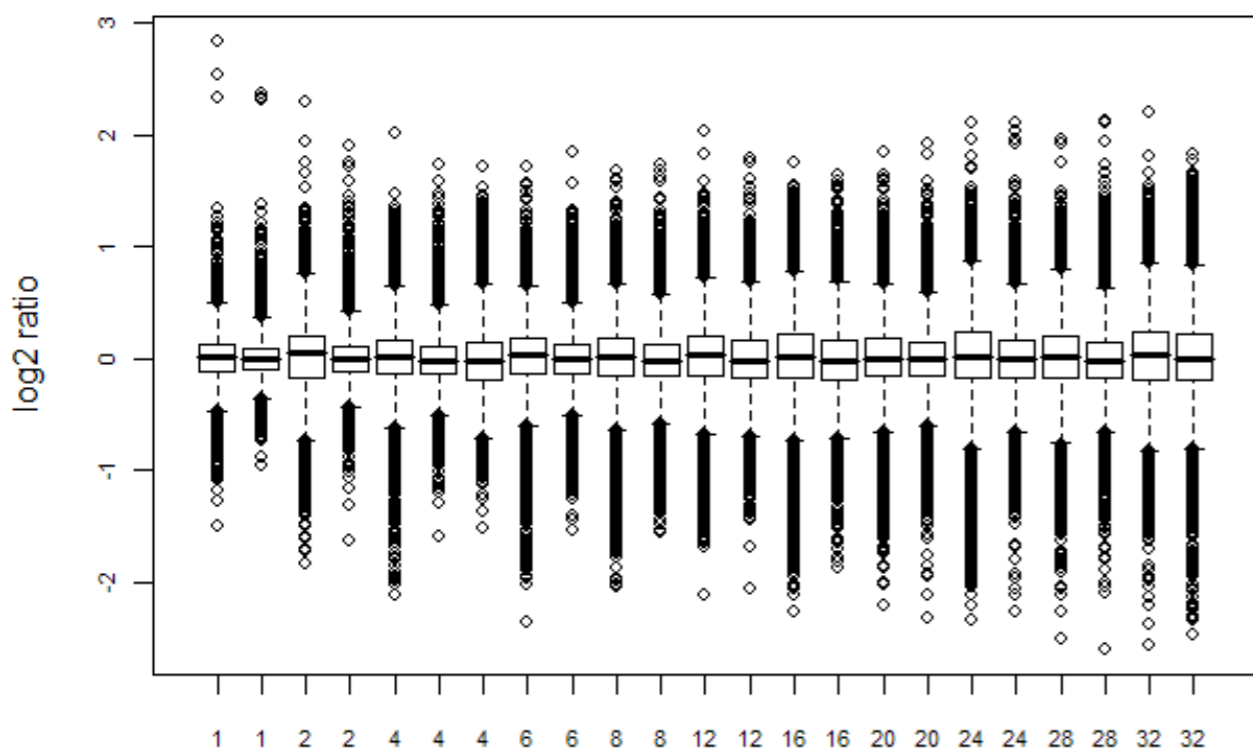

lumi

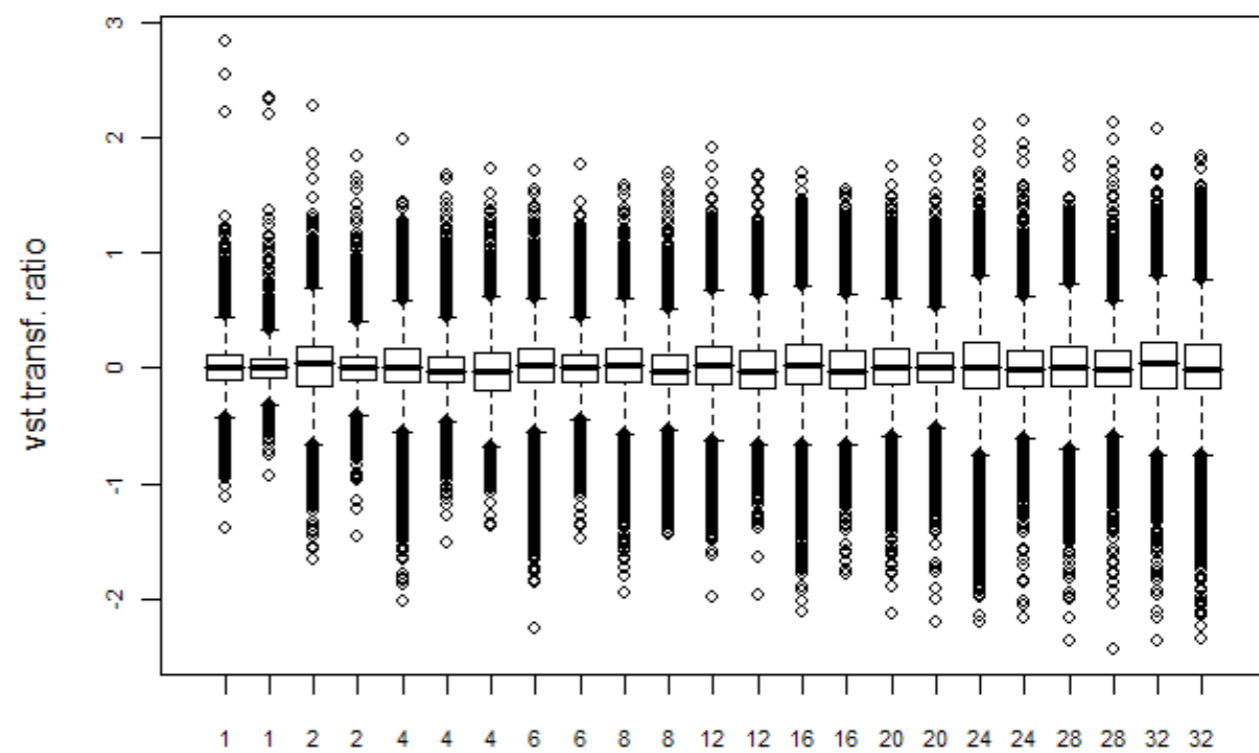

Supplement: Additional file 1: Normalization boxplots — Boxplots of normalized data after filtering and log-transformation. [file 1471-2105-9-S2-S12-S1.pdf]
